# Structural drivers of vulnerability to zoonotic disease in Africa

**DOI:** 10.1098/rstb.2016.0169

**Published:** 2017-06-05

**Authors:** Vupenyu Dzingirai, Salome Bukachi, Melissa Leach, Lindiwe Mangwanya, Ian Scoones, Annie Wilkinson

**Affiliations:** 1 Centre for Applied Social Sciences, University of Zimbabwe, Harare, Zimbabwe; 2 Institute of Development Studies, University of Sussex, Brighton BN1 9RE, UK; 3 Institute of Anthropology, Gender and African Studies, University of Nairobi, Nairobi, Kenya

**Keywords:** One Health, political economy, structural violence, zoonotic disease

## Abstract

This paper argues that addressing the underlying structural drivers of disease vulnerability is essential for a ‘One Health’ approach to tackling zoonotic diseases in Africa. Through three case studies—trypanosomiasis in Zimbabwe, Ebola and Lassa fever in Sierra Leone and Rift Valley fever in Kenya—we show how political interests, commercial investments and conflict and securitization all generate patterns of vulnerability, reshaping the political ecology of disease landscapes, influencing traditional coping mechanisms and affecting health service provision and outbreak responses. A historical, political economy approach reveals patterns of ‘structural violence’ that reinforce inequalities and marginalization of certain groups, increasing disease risks. Addressing the politics of One Health requires analysing trade-offs and conflicts between interests and visions of the future. For all zoonotic diseases economic and political dimensions are ultimately critical and One Health approaches must engage with these factors, and not just end with an ‘anti-political’ focus on institutional and disciplinary collaboration.

This article is part of the themed issue ‘One Health for a changing world: zoonoses, ecosystems and human well-being’.

## 1. Introduction

A common focus of One Health analyses is on the various economic and ecological drivers of zoonotic disease. This is often linked to maps of risk that guide intervention, where geographic ‘hot spots’ are identified, commonly in Africa [1,2]. Such depictions create a technical, ‘anti-politics’ [3] of One Health, where underlying political, social and cultural factors with a potential bearing on emergence of zoonotic diseases are hardly considered. One Health approaches of course recognize the intersection of different drivers, and espouse the close working together of veterinarians, medical professionals and social scientists [4]. But could it be that there are structural issues of a political economy nature—such as histories of ‘development’, ‘investment’ and ‘securitization’—that provide an underlying foundation for vulnerability to disease? Further, could it be that for better disease control strategies, these intersecting forces need to be thoroughly understood?

Craddock and Hinchliffe [5] have advised on the need and value of taking a political economy approach if we are to uncover the factors that truly make people vulnerable to disease in developing countries. Who gets sick and where, are not simply ecological or demographic outcomes. Often forces of a political and economic nature create disease, and more crucially, determine the manner of its management and control. Development interventions can displace people to marginal places, making them vulnerable to disease. Equally, security concerns may lead to a concentration of people in a specific locality as refugees or migrants, where disease can transmit easily. From this perspective, a political economy approach is necessary to deal with conditions that make people vulnerable to disease. It is not simply getting medical scientists, veterinarians, ecologists and social scientists together that tackles the pathologies of poverty and underdevelopment that generate vulnerabilities to disease.

This echoes a political ecology perspective that argues that political and economic forces are often behind societal vulnerability to hazards and risks [6], and linked to historical patterns of underdevelopment [7]. These political and economic forces can create ‘underlying generative structures’ of precarity [8]. For example, vulnerabilities may be generated by development projects designed to control communities for the benefit of national elites [9,10]; neo-liberalism with its emphasis on cutting public expenditure and leaving many without any safety nets [11]; privatization that excludes people from the commons [9,12]; and markets, which advance interests of big businesses while destroying smallholder agricultural livelihoods [13]. In short, a political ecology perspective sees vulnerability as primarily located in the contest between different groups and classes over access to resources [14]. Our argument therefore is that exposure to disease is crucially a function of historical, political and economic forces. Echoing Michael Watts [15], we argue that in their quest for accumulation or modernization, the state and private businesses can ‘produce’ disease conditions, and expose poor and marginalized people. In some cases, the state and business investors marginalize and impoverish people, confining them to conditions where disease is likely to emerge. Our argument also draws on Paul Farmer's [16] ideas on ‘structural violence’. Poverty and inequality are frequently central to infection dynamics, and zoonoses and other emerging diseases can be seen as pathologies of power and politics [17], requiring political intervention [18].

Such perspectives however have not been fully applied to examine the relationships between disease and society. The result is that our understanding of underlying drivers of diseases remains partial. In our framework, ecology, demography and livelihoods must be looked at in combination with the entrenched political and economic forces that are foundations of vulnerability. As the world desperately searches for solutions to deal with zoonotic diseases under the banner of ‘One Health’, the time is ripe to try this approach focusing on the political economy of vulnerability, and the underlying structural drivers.

Our insights derive from an examination of three zoonotic disease cases from the multi-disciplinary Dynamic Drivers of Disease in Africa project, which brought together epidemiologists, veterinarians, sociologists and rural communities. Our cases focus on trypanosomiasis (Zimbabwe), Lassa fever and Ebola (Sierra Leone) and Rift Valley fever (Kenya). The following sections explore how structural, political–economic drivers of vulnerability are important in all three cases, with implications for how a One Health response should be seen.

## 2. Trypanosomiasis and tsetse in Zimbabwe

Trypanosomiasis is a parasitic disease transmitted by tsetse flies. In Zimbabwe, the disease is concentrated in the Zambezi valley, including Hurungwe which is the case study area. In this district, each year there are reported cases of trypanosomiasis in both humans and livestock. Years of intense control has reduced the seriousness of the problem and today the disease is not prioritized by national control agencies. Recent studies indicate that flies are located in protected areas, valleys and some mountains with requisite climate and vegetation [19]. Similarly, cases of trypanosomiasis are found in areas that are close to forest patches that are accessed by people for a range of livelihood resources, including wildlife and non-timber forest products [20]. Epidemiological surveys reveal that livestock living near such patches are exposed compared with those in the cleared, more settled areas [21]. In Hurungwe, certain groups tend to be more vulnerable to disease than others. People reporting problems with tsetse flies are foragers, squatters, cattle herders and hunters; all groups who regularly enter the patch. How has this social and spatial configuration of vulnerability emerged in Hurungwe?

### (a) Modernization, conservation and development: creating vulnerability in diseased landscapes

To understand why some people living in some places in Hurungwe district are vulnerable to trypanosomiasis, we must take a historical political economy perspective. The following sections document how combinations of modernizing development and large infrastructure projects, the commercial expansion of capitalist agriculture and environmental businesses, and war and insecurity at different moments have both created new diseased landscapes and pushed people into them through displacement.

In a bid to modernize the Rhodesian colony and create a secure supply of electricity and to create affable places of nature and leisure for the benefit of colonial elites, the Rhodesian settler state built the Kariba dam in the 1950s [22]. Around this massive piece of engineering, national parks were established, including the Mana Pools in the north and Sapi Forest Areas in the northeast. At the same time, places that combined wilderness and sport hunting were designated including Hurungwe Safari area and Chewore Safari area [23]. As a result of the creation of Lake Kariba and the establishment of the wildlife zones, people were forced out and required to move away from their home areas alongside the river to occupy the forested lands to the south. Lake Kariba and accompanying developments displaced more than 15 000 Tonga and Korekore households. These groups were resettled in the modern day Hurungwe which was already tsetse infested and enclosed by wildlife areas, also full of tsetse [24].

To the north, people were also constrained by the expansion of commercial farmland on the plateau, where a post Second World War tobacco boom resulted in significant profits for white settler farmers [25]. Commercial farmers had lobbied for tsetse clearance operations to clear ‘white’ farmland of the fly. By the 1960s, Hurungwe was declared a native reserve district, and was accommodating increasing numbers of people in tsetse fly-infested forest areas, encircled by wildlife and white settler commercial farms. Displacements from other parts of the country grew from the 1950s when ‘land husbandry’ policies were implemented in the African reserves [26] and continued over the following decades as land hungry farmers sought new lands outside the crowded communal areas and spontaneously settled in the land-extensive, but tsetse-infested, Zambezi valley areas [27] Patterns of vulnerability changed dramatically in Hurungwe district in the 1970s as the country's war of liberation intensified as resentment of colonial land seizures, forced resettlements and taxation grew. The battlefront included the forests of the Zambezi valley, including Hurungwe [28]. During this period, the settler government reorganized villages with the aim of separating guerrilla fighters from supportive villagers [29]. People living in the forests and near protected areas were forced into protected and settled villages such as Chitindiva. This consolidation of villages meant that the ranges of wild animals expanded, including those carrying the disease [30]. With tsetse control operations ceasing because of the war, the tsetse belt expanded and trypanosomiasis became a major scourge in the late 1970s, and into the 1980s, following independence [31].

Following independence in 1980, major efforts were invested in tsetse control, with the European Union funded programme, the Regional Tsetse and Trypanosomiasis Programme (RTTCP), engaging in major control operations [32]. As in the colonial era, political-economic interests were important. Different lobbies argued that tsetse eradication would unlock agribusiness and tourism opportunities in the richly endowed Zambezi valley [33]. But vector eradication programmes were limited by others who saw the fly as the saviour of wilderness. Powerful conservation lobby groups saw commercial opportunities in these areas, protected by the tsetse fly. The result was the introduction of a consumptive wildlife utilization project, CAMPFIRE [34]. This project encouraged wildlife presence, including in settled areas, and this facilitated tsetse outbreaks affecting villagers living in CAMPFIRE areas.

From the late 1990s, the CAMPFIRE programme retreated with the collapse of the national economy, the rise in poaching and the resistance of local people dismayed at the lack of funds being shared with them. However, new initiatives were afoot that saw the potential of wild spaces for making profits. In 2012 Kariba REDD, a carbon project in Hurungwe, was established by a group of local investors (mostly white Zimbabweans who had lost out in the land reform or had been formerly part of the local hunting business). These investors joined hands with the local-level elites, declaring the district and adjacent areas as carbon areas that would generate millions of dollars from the market in a 30-year period [35]. To maximize potential returns from carbon, a giant buffer zone was created, where no settlement was permitted but only wildlife and forests. This again increased vulnerability because the project enlarged the habitat of tsetse fly and wildlife, and is as a result widely resisted by local people.

In the 2000s, the area saw a new wave of in-migration, as people were displaced as a result of land reform. This included in particular farm workers who had previously been employed on large scale, white-owned commercial farms on the Karoi plateau to the south [25]. Through complex micro politics, displaced farm workers were re-settled in the wildlife-rich buffer areas on the edge of the safari areas and national park, where they became extremely vulnerable to trypanosomiasis infection. Displaced migrants were extremely poor, living on the margins of existing communities, and without assets. They expanded agricultural production in small areas, encroaching further and further into the diseased landscapes. As with the earlier migrations from the dry south of the country, some have improved livelihoods, profiting from cotton and tobacco [36], as well as gardening along river banks. But at least initially many had to rely on other income sources. Plentiful natural resources provided the basis for new livelihoods with hunting (by men) and foraging (by women and children) central. Gardening, hunting and foraging, as well as just living in these areas, resulted in heightened exposure to tsetse fly and so risks of disease. There has also been a growth in informal, artisanal mining in the area. Many miners were retrenched from the nearby Alaska and Lynx mines, following the collapse of the economy [37], and they too have migrated to these areas, living in temporary huts along major rivers, where they pan for gold, including on Chewore River, which winds through the tsetse- and wildlife-infested areas to the Zambezi River. Migrants, often already highly marginal and poor, are thus forced to survive living and working in diseased landscapes, increasing their vulnerability to disease.

Over time, then, we see state-led development—from the colonial era to the present–generating particular visions of landscapes—modern, racialized, commercial, wild—that in turn generate disease vulnerability for certain people. We equally see the intersection of capitalist interests (the dam for electricity generation, hunting, tourism carbon) with elite politics, as alliances are generated to create landscapes of disease and vulnerability, focused on particular places and people. In sum, we see a ‘structural violence’ imposed that creates a political ecology of disease deeply implicated by political economy processes over time. Thus through a political economy approach, we are able to understand why trypanosomiasis disease areas exist and why certain groups of people end up being affected. In the next section we turn to examining Lassa fever and Ebola in West Africa.

## 3. Lassa fever and Ebola disease in West Africa

The 2014–2015 Ebola virus outbreak brought global attention to the Upper Guinea Forest of West Africa, a region with no known history of Ebola circulation. With over 22 000 reported cases and over 11 000 deaths [38], and with many more uncounted, this was the world's largest Ebola epidemic. With it came much commentary trying to explain its apparently new emergence. In accounts of its origins, the notion that rapid deforestation combined with poverty was newly bringing people, virus-carrying bats and infected wildlife into contact was pervasive [39,40]. Yet these accounts overlook much about the history, political economy and ecology of the region. We examine not just Ebola but Lassa fever, another viral haemorrhagic fever which has been recognized in the region since 1972. Traditionally thought to occur in ‘hyper-endemic’ zones in parts of Liberia, Sierra Leone and Guinea the disease is increasingly being detected outside these areas [41]. Both diseases have animal reservoirs—the rodent *Mastomys natalensis* for Lassa, and for Ebola, possibly bats, although this remains contested. Considering the two diseases together highlights revealing dimensions of the region's long-standing political–economic make-up and its vulnerability to outbreaks of epidemic-prone zoonotic disease.

From the fifteenth century or before, linked to climatic desiccation, the breakup of the Mali Empire, and European trading and slaving influences on the coast, Mande speakers settled in what is now the tropical forest belt in modern Sierra Leone, Liberia and Guinea [42]. Historical accounts suggest that this was already a mosaic of forest and savanna in climates that may have been historically drier than today [43]. Through centuries of settlement, farming and trade a forest-farm landscape has emerged which is characterized by central villages or towns surrounded by homegardens, agroforests and anthropogenic forest islands, beyond which lie upland swidden-fallow mosaics of rice fields and fallows. These are bisected by streams and swamps used for rice and vegetables.

Interactions with rodents, bats and other wildlife are a long-standing feature of life in this landscape. Rodents are routinely hunted for consumption by many sections of society [44]. *M. natalensis* is found mainly in homes, but also in swamps and fields, meaning people are routinely exposed to the Lassa virus in their domestic and agricultural activities, according to seasonal and gendered divisions of labour (see Leach *et al*. [45]). Rodents thrive in homes constructed in mud which is ideal for burrowing [46]. The story is much less clear for Ebola. While bats are a possible reservoir, there is little to suggest that bats and people have not been in similarly close contact, with bats long roosting in homes, trees surrounding villages, and caves in an agriculturally frequented landscape. Despite these uncertainties, what seems clear is that for both Lassa and Ebola the story is less one of rapid deforestation or human intrusion deeper into untouched forests, but more long-term cultivation and co-habitation in environments—swamps, gardens, mud homes and forest islands—favourable for these wildlife reservoirs.

As such, vulnerability to spillover of these diseases is likely to be a long-standing feature of life here. Yet, the increasing recognition of Lassa fever outside of its traditional ‘hyper-endemic’ zone in the east of Sierra Leone, and now the presence of Ebola too, provoke questions of why here and why now. The relatively stable relationship of people and animals described above suggests a number of scenarios. First, the increased disease incidence may be more a matter of improved recognition. Fever-like diseases have historically been poorly diagnosed in this region, with historical Lassa ‘hotspots’ more likely to be artefacts of biased surveillance [47]. The idea that Ebola is new to the region is contested by studies which have re-analysed old human blood samples collected in the region and found antibody evidence of Ebola infection prior to this latest outbreak [48]. A second explanation, pertinent for Ebola, is the emergence of the virus in local wildlife populations for the first time, rather than human contact with animals for the first time. A third possibility is that Ebola was brought by human movement from elsewhere in Africa. Combined with these possibilities though, is that some more medium term political-economic shifts may have made people more vulnerable either to spillover but especially to transmission by intensifying and altering patterns of settlement, human movement and land use.

For Lassa, where the majority of infections stem directly from rodents rather than human to human transmission [49], recent socio-economic dynamics which have altered housing and agricultural patterns have the potential to bring people into increased contact with rodents. Civil wars in Liberia and Sierra Leone caused population displacement, including urban areas and refugee movements back and forth into Guinea. A general trend, starting pre-war [50], but continuing since, is for smaller households. Down from groups of up to 50 people who had lived and farmed together, it is increasingly common for smaller family units based around a husband and wife to clear their own farm. The result is a larger number of smaller fields cleared, and possibly an increase in the number of gardens and small cash crop plantations in bush areas by these small households which are an important source of financial independence for both women and young men. Houses are smaller too, reflecting these changing agro-kinship arrangements. Instead of living in large co-habited houses, young men increasingly build their own modest houses as they become financially established, often initially from mud and stick which are cheaper materials. These conditions potentially create a multiplication of the ‘domestic’ spaces and peri-domestic areas in which *M. natalensis* live, and make pest control across villages or peri-urban areas more difficult to do comprehensively.

As rural farming opportunities have become more precarious and as institutions have downsized, men and women also migrate to areas where there are income opportunities but where living conditions are more insecure and potentially more prone to rodent infestations. These include mining areas, where settlements and mining camps are typically poorly constructed with mud house. The expansion of peri-urban areas on the fringes of larger towns, or where work may be available in commercial agricultural schemes, also create zones of potential vulnerability. To date, however, there is no clear evidence at the scale required to show an actual association with Lassa fever increase.

## 4. Political economy of outbreaks

Beyond the micro-effects of these political-economic changes on disease vulnerabilities are broader effects of systemic underdevelopment. The Sierra Leone–Liberia–Guinea border region had a central place in the Atlantic slave trade [51] and in vibrant trans-West African trading empires through the eighteenth and nineteenth centuries, as well as supplying labour and commodities (rubber, timber, cocoa and minerals) to British, French and Americo-Liberian powers [52,53]. While each country's colonial histories vary, they have in common the extraction of rich resources to the major benefit of foreign and national elites. Interventions often yielded little benefit to—and indeed exploited, dispossessed and did violence to—local populations.

Far from being a thing of the past, the Ebola outbreak has revealed starkly how these histories continue to shape patterns of development, producing vulnerability in the region and making it difficult to respond both to epidemics such as Ebola, and to a lesser extent to ongoing endemic diseases such as Lassa fever [54,55]. Most critical have been post-colonial development pathways which have fostered inequality and failed to address corruption or elite capture of resources, combined with a systematic underinvestment in state institutions precluding the establishment of resilient health systems, livelihoods and living conditions.

Independent governments continued colonial patterns of uneven development and resource extraction. In the 1970s, alluvial diamond extraction became the mainstay of Sierra Leone's patrimonial political economy of Siaka Stevens, notorious for its rent-seeking, corruption and related extreme inequalities [56]. In Guinea, Sekou Touré's 1958–1984 regime fostered a pattern of intrusive state socialism that concentrated resources in the hands of politico-ethnically favoured Manding elites [57]. In both countries this has fed into political systems predicated on patronage of one power base and the marginalization of others, at the expense of inclusive state institutions. Existing divisions were exacerbated during 1980s donor-led structural adjustment programmes which saw state resource flows squeezed.

Amidst present-day neoliberal capitalism, this pattern has continued, fostered by an extractive resource boom which ushered in stunningly high growth rates, up to 21% in Sierra Leone in 2013. However, this growth was not distributed nor has it been matched by effective governance and institution building. Several projects have been beset by major corruption scandals. For instance, the Federal Bureau of Investigations has alleged that former president of Guinea, Lansana Conté, and his now widow Mamadie Touré, accepted bribes for the development of mines, including Simandou the largest iron ore deposit in the world. Most recently, The Panama Papers leak has exposed offshore accounts belonging to Touré and used to move some of these bribes, and avoid tax on them [58]. Another boom area has been in the agriculture and forestry sectors, which has seen large-scale foreign investments in export food crop, biofuel and now carbon credit markets. Yet similar to mining, this has brought profit to foreign and local elites, largely at the expense of smallholder rights and livelihoods, undermining rural institutions [59,60].

These political–economic relations have also failed to build and sustain adequate health systems. Health systems have been systematically weakened by aid conditionalities and structural adjustment programmers which scaled back state spending on health in the 1980s and 1990s [61]; vertical donor programmes which focused only on particular populations or diseases, leaving more general care unimproved and corruption scandals [62]. Even before Ebola, many people decided against formal healthcare facilities, favouring instead traditional healers and informal vendors with their more personal approach and pluralistic understandings of disease and therapy [63], including for haemorrhagic fevers such as Lassa [64]. Poor training and hygiene in formal facilities contributed to the transmission of Ebola and to the deaths of large numbers of health workers.

A mix of conflict and limited opportunities in rural agriculture has seen the capitals of Liberia and Sierra Leone grow rapidly. Poorly planned, with limited sanitation and lacking essential services, these dense urban areas proved fertile ground for the virus [65].

Finally, the tangible ways in which entrenched inequality made people vulnerable to infection were combined with its corrosive impact on social relations. When it was most needed trust between local populations and their governments and responding aid organizations was elusive. Rumours that Ebola was manufactured to make money, or kill people, provoked widespread fear, violence and avoidance. The plausibility of these rumours is rooted in people's experiences of acutely unequal and deadly political economics, from slavery to modern day corruption, where extraordinary wealth has been generated for some at the expense of others [66].

## 5. Rift Valley fever in Kenya

Rift Valley fever (RVF) is a zoonotic disease transmitted by mosquitoes. It usually follows heavy rains, when mosquito eggs that had been lying dormant in the ground hatch. RVF cases in Kenya have intensified since the 1990s. In both the 1997–1998 and 2006–2007 RVF outbreaks, the initial epicentre was in the northeast of the country, where poverty levels are estimated at 70% of the population [67]. The most devastating outbreak occurred in 1997–1998 when approximately 27 500 infections occurred in Garissa, the largest ever recorded outbreak of RVF in East Africa [68]. The 2006–2007 outbreak resulted in widespread infections in the sparsely populated pastoral areas, with Garissa (31%), Ijara (22%) and Wajir (11%) reporting 60% of all cases countrywide [69]. During these RVF outbreaks, poor and marginal pastoral communities and those linked to associated value chains suffered the greatest impacts. Impacts resulted from both livestock and human mortalities, as well as restrictions on livestock movement and trade.

In the next section we unravel the multiple political and economic forces—including political marginalization and underdevelopment, irrigation and infrastructure investment, and conflict and securitization—that make pastoral areas and people vulnerable to these outbreaks.

### (a) Political marginalization and underdevelopment

Northeast Kenya, formerly North Eastern Province and the Northern Frontier District, is a historically neglected region. The area was denied development as a punitive measure for its secessionist commitments, having openly declared a desire to be part of the newly formed Somali Republic in 1960 [70]. These measures resulted in *Shifta* wars of 1963 [71]. From that period on, the region was under martial law until 1982. During that period, entry into the region was restricted to only civil servants and members of the Security Forces and since government's energies and resources were largely directed towards security and the maintenance of law and order, the region has suffered marginalization and underdeveloped investment. To this day, several roads in Northeastern Kenya are generally impassable especially during the rainy season, when RVF disease outbreaks occur, making state responses slow or impossible. Key services for human and veterinary health are limited, making disease control and treatment difficult. A testimony from a community leader who suffered livestock loss because of inadequate health care facilities is illustrative:

… the service rendered to livestock is very scarce, too little, too little, … ; we don't have a medical officer or veterinary officer, we do not have a vaccine store here, there is no pharmacy for drugs… There are just animal health workers. In our place we have got only one person, who cannot manage all community. (Male KII, Sangailu Ijara sub-county, 18/09/13).

A government official who was stationed at Ijara during the 2006–2007 RVF outbreak, observes:

There were not enough officers to help us. You could not get transport to the place. The road was impassable so we were even using helicopters to get to those areas in need of help. Many people were affected and could not all be treated in the small hospital which had only three rooms. There were no adequate drugs. So we had a hard time managing the patients. (Male KII, Masalani Ijara sub-county, 17/09/13).

It is not only underdevelopment and marginalization that has produced RVF; sometimes it is inappropriate development by the state that has led to vulnerability. Nowhere is this more evident than in the case of irrigation schemes.

### (b) Irrigation and infrastructure investment

In a bid to diversify livelihoods, which in the frontier area have been centred on a fragile pastoral economy, the Kenyan government has launched various development projects, in alliance with private sector players [72]. A modernization narrative dominates that sees large-scale investment in irrigation schemes and infrastructure, including hydroelectric dams, as central to development. For example, TARDA (Tana and Athi River Development Authority) was formed in 1974 to spearhead development along the Tana and Athi rivers [73]. In recent years, TARDA has facilitated investment by the private sector, in order to develop dams for hydroelectric power on the upper Tana. These dams have a total catchment coverage of about 138 000 square kilometres, and yield over 400 MW of electricity [74].

The ambitious Tana River irrigation schemes, for example, have been established in areas traversing former pastoral areas. These irrigation schemes created a new watery landscape hence modifying the transmission of vector-borne diseases, including RVF and malaria. Conditions for year-round breeding of mosquitoes were created through perennial canals feeding the irrigation plots. Mosquito populations were also encouraged by swamps and damp areas created by the overflowing of canals and in irrigated fields. Pastoralists were allocated plots in the irrigation schemes, however, as last to get plots, pastoralists were located at the tail-end of irrigation schemes, where stagnant waterways are frequently found [75].

The building of dams and irrigation schemes also put pressure on the riverine areas, intensifying conflicts between pastoralists and agro-pastoralists [76]. This promoted ethnically rooted animosities, with farmers and pastoralists violently clashing over water and pasture [77]. These conflicts stalled rural development and limited access to public health and veterinary services. When RVF broke out in 2006–2007, limited access to services meant that impacts were increased [78].

Many of these investment projects, dating from the post-independence era and accelerating recently, have disrupted pastoral movement, restricting use of dry season grazing areas along rivers, with limited compensation [79], Additionally, migrants have been attracted to work at the hydroelectric plants and irrigation schemes [80], and today there are fast- growing settlements competing with pastoral livelihoods. Pastoralists must make use of limited grazing, often on irrigation schemes where mosquitoes are now present throughout the year. This increases vulnerability to RVF, which has become endemic. Higher concentrations of animals in these areas means that disease transmission from one herd to another is high, which in turn can lead to increased human RVF infections [81].

### (c) Conflict and securitization

From the *Shifta* wars to the present, the border regions of northeast Kenya have been a zone of conflict. Ijara district finds itself furthermore marginalized for being far from its regional administrative centre Garissa, and for bordering Somalia where on the other side we find Al-Shabaab, a Jihadist group's stronghold [82]. Reportedly, military bases have been established in the Boni forests [Muriuki] which are an important source of grazing for pastoral communities [83]. According to one pastoralist in Sangailu, pastoralists have always made use of the area to escape from disease:

The movement of livestock matters to the people; when disease breaks out we go grazing as far as the coast. Later when trouble breaks out there, we drive our animals back to Boni Forest. Currently, we have our animals in Coastal Province where there is no disease. We used to retreat to Boni Forest as well. (Male KII, Sangailu, Ijara sub-county, 18/09/2013)

In a region where RVF is inevitable on account of ecological factors, indigenous mechanisms to deal with its effects are crucial. Mobility is essential in pastoral systems [84], and conflicts can prevent movement to key resources, such as the Boni forests.

In 2015 the Kenyan Security forces launched military operations against Al Shabaab in the forests [85], prohibiting all human and livestock presence. The militarization of the forests has denied pastoralists a traditional place to hide in the event of a disease outbreak, and reduced access to grazing, undermining already vulnerable pastoral livelihoods [86]. This has forced pastoralists to make use of the disease-vulnerable areas as safe refuges are now prohibited areas.

Vulnerability to RVF emerges out of structural, political-economic factors that have changed pastoralist livelihoods in the region, due to long-term marginalization, new investment projects and conflict and securitization. We can only understand disease vulnerability with reference to these underlying structural processes, which influence the spatial dynamics of transmission and infection, and so who is affected and where.

## 6. Conclusion

In this paper we have looked at three cases of zoonotic disease—trypanosomiasis, Rift Valley fever and Ebola/Lassa fever—in different parts of Africa, and explored how patterns of vulnerability are constructed through structural political–economic factors over time. These diseases do not just emerge in particular localities through immediate drivers of climate, ecology, demography and livelihood practices, but they emerge through long-term forces linked to political, commercial and security interests. ‘Structural violence’ and political ecologies intersect to generate vulnerabilities for particular people. In Zimbabwe, for example, investments in dam infrastructure and the expansion of commercial farming squeezed populations into increasingly marginal, disease-prone settings, with disease risks exacerbated by commercial interests in wildlife hunting and carbon sequestration. In Sierra Leone, although people have lived in close contact with wildlife reservoirs, recent development patterns are making people more vulnerable to high impact spillovers. For Lassa the trend towards smaller household and farming units is providing increasingly comfortable niches for the rodents who carry the disease, and limiting opportunities for collective responses to pest control. For Ebola—and other epidemic-prone diseases—neglected health services combined with growing gaps between elites and governments who follow non-inclusive development strategies, and the rural and urban poor whose trust and livelihoods are undermined by them, has compromised responses to outbreaks. In Kenya, long-term underdevelopment of pastoral areas has resulted in exposure to disease and limited outbreak response and treatment facilities, while alliances between the state and private sector have reconfigured pastoral landscapes with new dams and irrigation schemes, excluding pastoralists from dry season grazing reserves and changing the disease ecology, further enhancing vulnerability to those making use of irrigation areas for grazing and farming. Furthermore, conflict and the militarization of the area has generated a ‘securitized’ landscape that restricts mobility and removes areas to escape from disease.

Our cases show how disease outbreaks and transmission dynamics are affected by historical, political–economic processes, rooted in structural relations of politics and interests [16]. This conclusion has huge implications for One Health approaches. If a One Health approach is to respond to the political ecology of vulnerability, and deeper processes of ‘structural violence, it must move beyond a limited focus on proximate drivers of disease to look at underlying processes. It must also move beyond a focus that emphasizes only the integration of multiple disciplines at various scales to ensure health for people, animals and environment, and tackle the wider political–economic conditions that give rise to disease in the first place. For a One Health approach to be effective it must reject an anti-political, technocratic approach, and embrace a wider analysis of historical political economy and ecology, with an appreciation of the structural drivers of disease. This is challenging, as most One Health practitioners come from the technical disciplines of medical and veterinary science or ecology, and social and political aspects are often not central [87,88]. However, as our cases show it is essential to debate how disease landscapes and differentiated vulnerabilities are generated from different political economy contexts over time. A wider debate that puts politics at the heart of One Health practice must be envisaged, which facilitates dialogue among actors—different groups within the state, the private sector and civil society—to explore contrasting visions, interests and motivations, and the implications for disease vulnerability for different groups of people. This will not be easy, as entrenched, incumbent interests exist that continue to generate vulnerabilities and reinforce marginalization, as we have shown across the three cases. Fundamental conflicts may exist between different pathways of development, and with these contrasting interests in the future of an area. However, if we are to understand the structural drivers of vulnerability to disease in Africa [89], and respond to the threat of zoonoses, these conflicts and trade-offs must be at the heart of any One Health analysis and must be central to the operationalization of One Health in what are inevitably highly charged, conflictual political settings.
